# Medical Students and Faculty Perceptions About Online Learning During COVID-19 Pandemic: Alfaisal University Experience

**DOI:** 10.3389/fpubh.2022.880835

**Published:** 2022-06-23

**Authors:** Shoukat Ali Arain, Mahnoor Ali, Lana Arbili, Muhammad Faisal Ikram, Junaid Kashir, Aamir Omair, Sultan Ayoub Meo

**Affiliations:** ^1^Department of Pathology, College of Medicine, Alfaisal University, Riyadh, Saudi Arabia; ^2^College of Pharmacy, Alfaisal University, Riyadh, Saudi Arabia; ^3^College of Medicine, Alfaisal University, Riyadh, Saudi Arabia; ^4^Department of Anatomy, College of Medicine, Alfaisal University, Riyadh, Saudi Arabia; ^5^Department of Medical Education, King Saud bin Abdulaziz University for Health Sciences, Riyadh, Saudi Arabia; ^6^Department of Physiology, College of Medicine, King Saud University, Riyadh, Saudi Arabia

**Keywords:** COVID-19, medical education, online learning, perception, virtual learning

## Abstract

**Background and Objectives:**

Off-campus online learning methods abruptly increased and gained popularity during the COVID-19 pandemic. Previous studies have highlighted the limitations of online learning mode; however, further studies on the experiences of medical students are needed. This study aimed to investigate the preclinical medical students and faculty members' experiences with online education and learning.

**Subjects and Methods:**

In this cross-sectional study, data were collected using convenience sampling. Two hundred nine students and 13 faculty members who participated in the online courses offered during the spring semester of 2019–2020 completed an online questionnaire. A 30-item questionnaire for the students and a 25-item questionnaire for the faculty were used in this cross-sectional study.

**Results:**

Overall, 30% of the student sample was satisfied; importantly, high-achieving students (GPA > 3.5) were less satisfied (25 vs. 32%; *p* = 0.006). Satisfaction was also low (35%) for student-faculty interaction opportunities. About half of the student sample agreed that small-group interactive sessions would improve learning (53%). The most favored course format was the blended mode (43%), followed by traditional (40%) and online modes (17%). Six out of 13 (46%) faculty members were satisfied with their online experiences. Most of them found virtual teaching applications convenient (77%). Conversely, few faculty members agreed to interact effectively (54%), while 69% favored a blended format.

**Conclusions:**

The level of satisfaction in fully online courses offered during the COVID-19 pandemic remained low, especially among high-achieving students. Both students and faculty favored the blended format for future purposes. Small group active-learning strategies and web-based interactive tools may facilitate engagement and student-faculty interactions.

## Introduction

The coronavirus (COVID-19) is a highly infectious contagion. To limit its spread, social distancing protocols were implemented, including the closure of educational institutions. Consequently, pedagogical approaches suddenly changed with a remarkable rise in completely off-campus distance learning methods using digital platforms (1).

Distance learning refers to the provision of access to learning for those geographically distant. In recent times, it includes the involvement of technology-supported (online) learning that could either be synchronous, asynchronous or both methods. Synchronous learning refers to all the types of learning that take place in real-time over a set class schedule. Asynchronous learning, however, does not require real-time faculty-learner interactions; content is available for students to access at their own convenience. Furthermore, while a virtual learning program can be totally online, it can also be structured as a combination of both online and face-to-face campus learning methods, termed blended learning (2, 3).

Online learning aims to construct knowledge effectively by providing opportunities for learners to engage with the learning material through spaced repetition, learning context, and faculty feedback. This method should facilitate faculty-learner and learner-learner communication and provide opportunities for faculty members to support learners (4).

Notwithstanding the fear of some faculty members, online learning has been found as an effective modality of learning in different educational settings (5, 6). It has the advantages of being convenient and flexible for the learners and helpful in the continued professional and academic qualifications of those who do not wish to leave their workplace (7, 8). Conversely, despite the increasing enrolment in blended and online courses, student preferences and engagement with online material have been variable (9, 10). It is recommended that faculties try innovative online approaches to actively engage learners through interactive teaching-learning activities, formative assessments, and feedback (11).

Integration of distance online learning into the medical curricula has been minimal. The online mode offered critical continuity in medical education during the pandemic that had disrupted conventional teaching and education (12, 13). However, the unanticipated implementation of social distancing measures and subsequent lockdown led to a sudden rise in completely off-campus online learning pedagogies. The inadequately planned courses offered during this medical emergency differed from well-thought-out distance learning experiences. The provided courses could not be adjusted to accommodate the needs of optimal online learning. Though that may be, precious lessons can be learned to reshape medical education, as it seems likely that some of the precipitous changes brought to the field may persist beyond the pandemic (14).

To ensure that the implementation of distance learning remains effective and achieves inclusivity, various aspects of online learning need to be prioritized, including the use of digital tools and platforms, blending of appropriate approaches, rules of distance learning, and monitoring of learners' progress. Therefore, this study examines the perception held by preclinical medical students and faculty regarding various online courses domains that may help adapt appropriate pedagogical approaches to augment the online learning experience.

## Subjects and Methods

### Institutional/Study Context

The College of Medicine, Alfaisal University Riyadh, Saudi Arabia, offers a spiral MBBS curriculum with a gradual shift from basic to clinical disciplines, published previously (15). The curriculum is completed in ten semesters spanning over 5 years. The curriculum of the first 3 years consists of basic and preclinical subjects, including anatomy, physiology, pathology, and pharmacology, with an introduction to the clinical disciplines. The courses are offered in the form of integrated organ-system-based modules. Both active and passive teaching-learning strategies are in place, with problem-based learning (PBL) and team-based learning (TBL) constituting major active learning strategies.

With the implementation of social distancing measures in March 2020, an emergency decision was made to limit all teaching-learning activities to virtual sessions. The IT department of the university conducted workshops to train faculty members in the use of open-source web conferencing platforms and were encouraged to give synchronous live streaming sessions. However, some faculty members recorded a voiceover narration in PowerPoint presentations. All the live sessions were also recorded, and all curricular content was made available through the university's intranet platform (Moodle) for asynchronous learning.

Virtual instructions consisted of live lectures through web conferencing platforms or recorded PowerPoint presentations with narration. Videos demonstrating gross anatomy structures were recorded and uploaded on the intranet. Toward the end of each week, interactive discussion sessions were conducted based on queries sent by the students, along with an explanation of the important concepts. Small group sessions to facilitate PBL and TBL could not be undertaken, and their learning objectives were covered in whole-class lectures instead.

### Survey Design

In this cross-sectional study, data were collected using convenience sampling. Preclinical students from year 1 to 3, and faculty members who were involved in online courses offered during the spring semester of 2020, completed an online questionnaire. All the students and faculty members who filled out the survey were included in the study. The study was approved by the Institutional Review Board, Alfaisal University, Riyadh, Saudi Arabia (Ref # IRB-20049).

A 30-item questionnaire for the students and a 25-item questionnaire for the faculty were created on Google Docs, and a link was sent through the university's official group email address. The questionnaire consisted of multiple-choice questions, Likert scale questions, and open-ended questions. Data on demographic variables, 'students' self-reported GPA, preferences for the course formats, and experience with the online courses were also collected. Participants were informed about the aim of the study at the beginning of the survey. Consent to the survey was inferred from the completion of the survey. All the data were collected anonymously and have been reported cumulatively.

### Statistical Analysis

The data on the perception of online courses were collected on a 5-point Likert scale as frequencies (strongly disagree being one; strongly agree to be five). Likert scale responses “agree” and “strongly agree” were grouped into “agree.” Similarly, “disagree” comprised all “disagree” and “strongly disagree” responses on the Likert scale.

Data were analyzed using the Statistical Package for the Social Sciences version 27 (SPSS Inc., Chicago, IL). Percent agreements for different survey items were calculated, and comparisons of the survey items were made through the Chi-square test. In all analyses, a *P*-value of <0.05 was considered significant. Open-text comments were reviewed independently by two authors (SAA and AO). Repeating patterns were identified as themes. Similar comments that appeared across the data set were coded under the identified themes. Later, themes and indicative comments were compared to reach a consensus.

## Results

Out of a population of around 600 students and 25 faculty members, 209 students and 13 faculty members responded. The distribution of the responding students according to the demographic variables is depicted in Table 1. An appropriate allocation of representation across all three preclinical years is evident, and most of the participants had a realistic experience with online courses.

**Table 1:** Demographic data of participating preclinical students (*N* = 209).

**Parameter**	**Number**	**Percent**
Gender
Male	66	31.6
Female	143	68.4
Year of study
Year 1	80	38.3
Year 2	42	20.1
Year 3	87	41.6
Ethnicity
Saudi	64	30.6
Arab (Non-Saudi)	105	50.2
Non-Arab	40	19.1
Self-reported GPA (out of 4)
Upto 3.5	124	59.9
More than 3.5	83	40.1
Number of courses taken online
Upto 4	81	38.8
More than 4	128	61.2

*GPA, grade point average*.

### Course Rating on the Likert Scale

The perception of students is summarized in Table 2. Only 30% of the students were satisfied, while 47% of the student sample was not satisfied with their online learning experience. However, slightly more than half of them agreed that lectures (58%) and laboratory sessions (58%) were well-aligned with the learning objectives. Concerning lecture formats, the opinion was divided; 33% enjoyed the live sessions, while 39% preferred recorded PowerPoint presentations. Similarly, only 36% agreed that live anatomy demonstration sessions were engaging. Satisfaction was rated low (35%) for the availability of adequate opportunities to interact with the faculty as well, while 54% agreed that the faculty responded to queries adequately. About half the respondents agreed that small group interactive sessions will improve learning (53%) and can be virtually conducted (45%). Notably, 59% of students agreed to the idea of face-to-face contact being necessary for learning.

**Table 2:** Perception regarding online learning experience based on the Likert scale responses (*N* = 209)^*^.

**Survey items**	**Agree**	**Neutral**	**Disagree**
I am satisfied with my online learning experience	62 (30)	49 (23)	98 (47)
Online lectures were aligned with learning objectives	120 (58)	54 (26)	34 (16)
Anatomy demonstration/lab sessions were aligned with learning objectives	107 (58)	52 (28)	25 (14)
I enjoyed live sessions the most (Zoom, BigBlueButton, etc.)	69 (33)	61 (30)	77 (37)
I prefer voice-over PowerPoint lectures	82 (39)	56 (27)	71 (34)
Anatomy demonstration/lab sessions were engaging	66 (36)	66 (36)	51 (28)
There were adequate opportunities to interact with the faculty	73 (35)	67 (32)	69 (33)
Overall, faculty responded to queries adequately	113 (54)	65 (31)	31 (15)
Inclusion of virtual small group interactive sessions (e. g., PBL, TBL) will improve learning	110 (53)	54 (26)	44 (21)
Small-group interactive sessions can easily be conducted virtually	94 (45)	49 (24)	65 (31)
Face to face contact between faculty and students is necessary for learning	124 (59)	42 (20)	43 (21)

^*^There are a few missing responses for some rows; data are shown as n (%), and the percentage is rounded-off to whole numbers.

### Key Facets of Course Format

**Figure 1:**
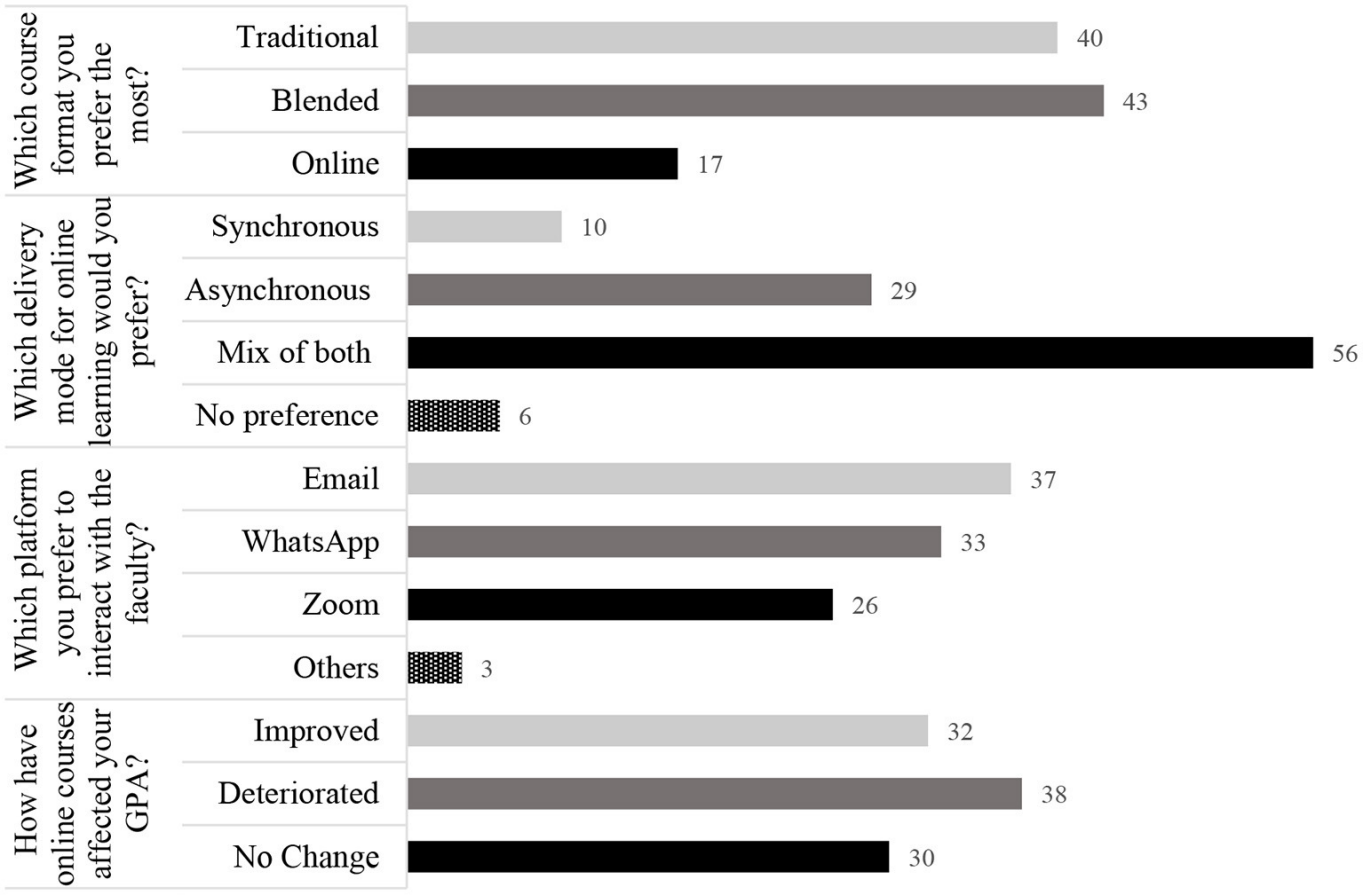
“Students” preferences for key components of course formats and self-reported effect on GPA (*N* = 209). GPA: grade point average. Data are shown as a percent (rounded-off to whole numbers).

Students' opinion regarding key facets of course format and the effect on their self-reported GPA is shown in Figure 1. Blended was the most favored (43%) format, followed by the traditional face-to-face (40%) course format, the purely online course format being the least liked of all (17%). As a mode of content delivery for the online component of the curriculum, more than half (56%) of the respondents favored a mix of both synchronous and asynchronous learning methods, and more than a quarter (29%) preferred only asynchronous learning. To communicate with the faculty during online courses, opinion was distributed almost evenly among preferences for email, WhatsApp, and Zoom. Slightly more than one-third of students (38%) reported that their GPA deteriorated, while for the remaining 62%, it either improved or did not change.

### Comparison of Low-GPA Groups and High-GPA Groups

When compared for choices based on low-GPA and high-GPA groups (Table 3), a significantly low number of high-achieving students were satisfied with their online learning experience (25 vs. 32%; *p* = 0.006) and accordingly reported a deterioration in their GPA (29 vs. 52%; *p* = 0.004). In congruence with the same, many students with high GPAs favored face-to-face contact (71 vs. 53%; *p* = 0.021).

**Table 3:** Preferences for online learning based on low (*N* = 124) and high (*N* = 83) GPA groups.

**Survey item**		**GPA ≤3.5**	**GPA >3.5**	***P*-value**
I am satisfied with my online learning experience.	Agree	40 (32)	21 (25)	0.006*
	Neutral	36 (29)	12 (15)	
	Disagree	48 (39)	50 (60)	
Which course format do you prefer the most?	Traditional	46 (37)	38 (46)	0.14
	Fully online	26 (21)	9 (11)	
	Blended	52 (42)	36 (43)	
**Which of the following formats would you prefer for online learning?	Synchronous	14 (11)	6 (7)	0.59
	Asynchronous	38 (31)	22 (27)	
	Mix of both	66 (53)	49 (59)	
	No preference	6 (5)	6 (7)	
Face-to-face contact between faculty and students is necessary for learning	Agree	65 (53)	59 (71)	0.021*
	Neutral	30 (24)	10 (12)	
	Disagree	29 (23)	14 (17)	
How have online courses affected your GPA?	Improved	45 (36)	22 (26)	0.004*
	No Change	43 (35)	18 (22)	
	Deteriorated	36 (29)	43 (52)	

^**^Synchronous (live teaching); Asynchronous (recorded or prepared to learn material available to students at their convenience); Mix of both (Mix of synchronous and asynchronous). Data are shown as n (%). The percentage is rounded-off to whole numbers.

### Open-Text Comments

Response to open-ended questions is summarized as themes with their indicative statements, as shown in Table 4. Common themes include appreciation for the availability of recorded curricular content on the intranet and the comfort of attending from home, resulting in convenience and flexibility of time management. However, in the opinion of the students, engagement and interaction with faculty needed improvement. Interaction with friends and faculty, taking notes during sessions, the comfort of staying at home, and a desire to obtain a good GPA were common motivating factors for learning during the offered courses.

**Table 4:** Open c-text comments are arranged as common themes along with representative statements.

**Theme**	**Indicative statements**
**Which aspect(s) of virtual courses worked especially well?** Recorded live lectures and PPP with voiceover (30)* The flexibility of time management (14) Live sessions (13) Attending from home (15)	I could watch the recorded lecturers whenever I got the chance The recorded lectures allowed us to pause, rewind, forward, etc No mandatory attendance - The fact that I can learn at my convenience There was no need to attend the lecture at an exact time, which gave me freedom and the ability to organize my week The option of attending live sessions and having recorded lectures to return to I liked online classes because I had plenty of time to manage my studies There was the benefit of saving time as there was no commuting time to the university
**Which aspect(s) of virtual courses can be improved?** Interaction with faculty (30) Laboratory/demonstration session (8) Small group sessions (9) Faculty's training in the use of technology (5)	Sometimes it felt like reading off PowerPoints, which was not engaging Keep students engaged (pop questions, dividing topics, short videos, exercises to facilitate engagement) “Communication between doctors and students for example, doctors can have online office hours for a meeting on zoom Lab sessions were difficult to grasp online Conducting PBL and TBL will improve the students learning and outcomes Training faculty more about using technology and avoiding problems such as connection issues (voice suddenly cutting out, or the image freezing)
**How did you keep yourself motivated/focused during virtual learning?** Interaction with friends and faculty (11) Taking notes during sessions (10) Scheduling the activities and comfort of staying at home (14) By focusing on GPA/grades (13) I couldn't; it was difficult! (19)	The more interactive, the more it is easier to stay focused. Group studying virtually. Come on! I'm in my house in my pajamas, so I will not be motivated to study the lecture! I had extra time to exercise and balance my studying schedule to keep me focused. I was eating snacks and highlighting essential things in the lecture with the doctor. It was very hard, but I tried setting a routine and keeping a schedule. I would listen to the recordings later when my house was quieter, and I could focus better. Maintaining a high GPA motivated me. Virtual courses were great, but we missed the class interaction so much.

^*^Values are the number of similar comments.

### Faculty Feedback

**Figure 2:**
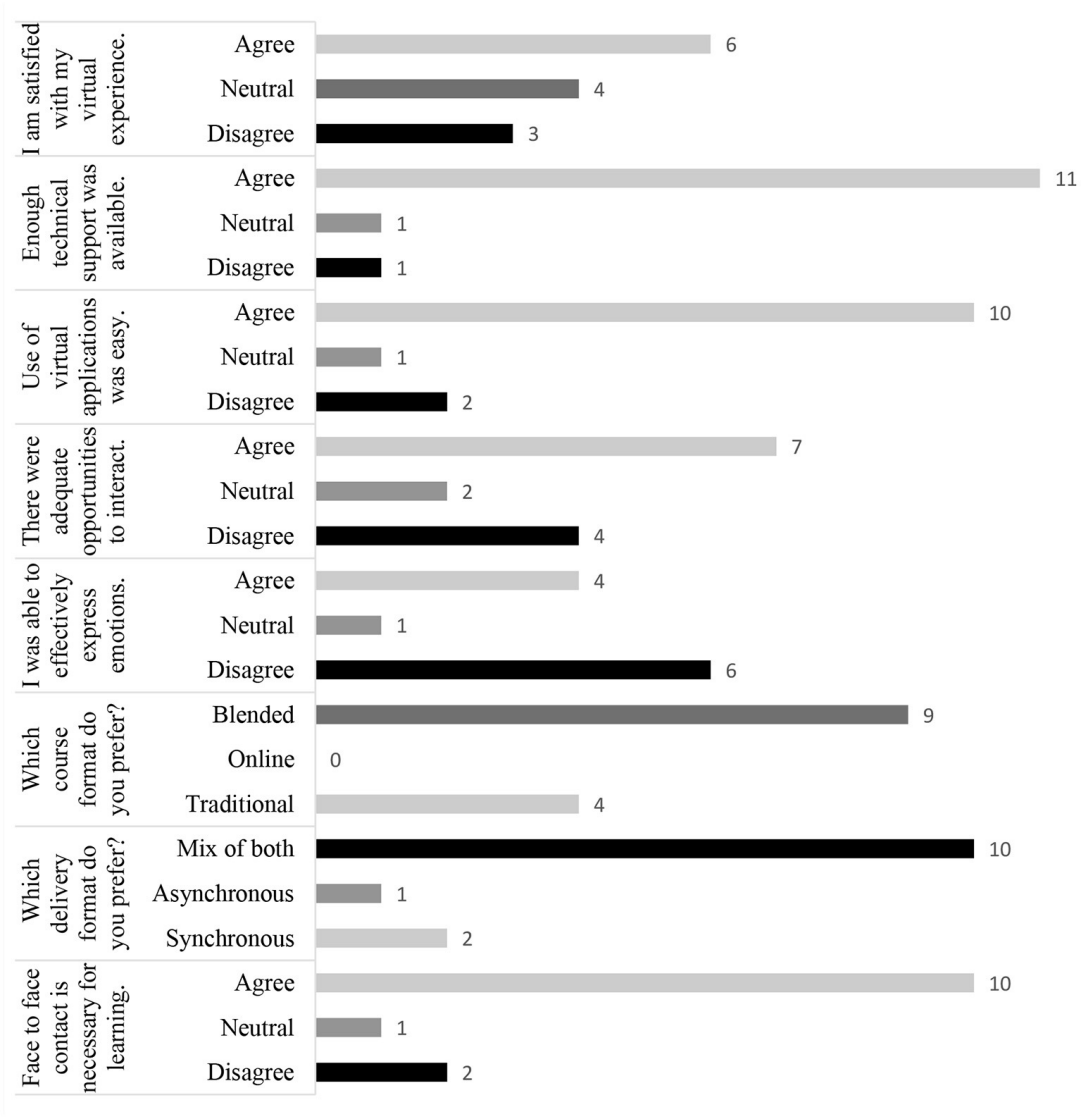
Response from the faculty (*N* = 13). Data are shown as the number of responses.

Out of 25 faculty members involved in teaching e-learning courses, 13 responded to the questionnaire (Figure 2). Six out of 13 (46%) faculty members were satisfied with their online teaching experience. Most of them were satisfied with the availability of technical support (85%) and found the use of virtual teaching applications convenient (77%). Although 54% of the faculty members agreed that there were adequate opportunities to interact with the students, only 31% agreed that they could express their emotions well-over these engagements. A majority of the faculty members (77%) favored face-to-face teaching, especially for anatomy demonstrations and laboratory sessions, as elaborated in the open-text comments. However, just as in the case of students, blended was the most favored format (69%), with a mix of both synchronous and asynchronous delivery modes for the online content (77%).

## Discussion

The outbreak of COVID-19 abruptly changed pedagogical approaches with the need for completely off-campus online learning. Although technology provided the opportunity for a swift switch to online teaching, emergency remote teaching and not well-planned online courses were offered. However, educational institutions may benefit from this opportunity to learn to devise inclusive virtual curricula and accelerate the reforms made in online learning.

In this study, only one-third of the students were satisfied with their online learning experience. We observed that the findings documented in our study are consistent with results from previous studies (16, 17). Hanafy et al. (18) also found that students favored conventional over online teaching. However, in other studies, students' perceptions positively preferred online courses (19). Our cohort reported major engagement and student-faculty interaction issues, consistent with the recent reports (20–22). Data on fully online preclinical courses in medical education prior to COVID-19 are scarce. However, comparative studies on small courses observed enhanced engagement in synchronous and asynchronous virtual formats compared to traditional on-campus learning (23). Still, the traditional face-to-face format was preferred for student-faculty interactions (24). Possible reasons for our cohort's lesser satisfaction and engagement may be attributed to some COVID-associated negative psychosocial outcomes (25, 26) and the conducting of fully online courses for the whole semester.

It was interesting to note that high-achieving students were significantly less satisfied. Accordingly, a higher number of students with high GPA reported deterioration in their GPA and favored face-to-face contact in their learning methods. In a similar study conducted in a university by students Owston et al. (27), the high achievers were the most satisfied with blended courses and preferred them over fully face-to-face or online format instead of the low achievers who preferred them face-to-face instructions. High achievers were less satisfied in our cohort, possibly since we offered fully online courses. Additionally, we classified high achievers based on their cumulative GPA and noted that GPA deterioration was significantly higher in this group in the offered online courses. Opinion was divided almost evenly among high achievers for their blended and face-to-face formats preferences.

Collectively, in our cohort, around two-thirds of students and faculty members favored either blended or online courses, while slightly over one-third preferred a traditional face-to-face course format. Likewise, Su et al. (19) concluded that flipped classroom teaching could encourage student-centered independent learning while using online courses.

In agreement with the other studies, our cohort reported several advantages, including the availability of all the recorded learning material on the intranet, saving commuting time, and the freedom to learn at their convenience and pace (25, 28). Students also provided various suggestions to improve engagement, such as pop questions, short videos, and practical exercises. Furthermore, they suggested including small group learning activities (e.g., PBL, TBL) to improve learning and student-faculty interactions, as indicated by others (17).

Unlike other studies (21, 29), our cohort did not report any significant problems with the availability of infrastructure or technical issues with technology and the internet. This may be due to our students already being familiar with online technologies in a web-based assessment platform (ExamSoft, Dallas, USA) and electronic-PBLs (30, 31).

### Study Limitations

A limitation of the study is that the offered online courses resulted from an abrupt transition due to a medical emergency and thus were radically different from well-planned distance learning experiences. Moreover, negative psychological outcomes of the current traumatic conditions may have affected the perception of the participants about the courses. However, this compelled use of online technologies provided a unique opportunity to learn for the future. Besides, due to a low number of participating faculty, meaningful comparisons could not be performed based on the demographic information and experience of the faculty (which may have had an impact on the perceptions of the faculty themselves). Studies should be carried out with the planned online course content to evaluate the role of virtual clinical training in clinical years and the outcome measures of direct and indirect student learning.

## Conclusions

The COVID-19 pandemic has compelled the medical fraternity to inculcate online learning practices in medical education. Although satisfaction with fully online courses during the COVID-19 pandemic remained low, especially among high-achieving students, the blended format was favored by both students and faculty for future needs. The students and faculty preferred the blended course format with the inclusion of both synchronous and asynchronous delivery modes for the online component. Measures to enhance ‘students' engagement and interaction with the faculty should be considered in the future planning of online learning experiences. The incorporation of specific face-to-face components, the inclusion of small group active-learning strategies, and the use of interactive tools over web conferencing platforms may facilitate engagement and student-faculty interactions.

## Data Availability Statement

The original contributions presented in the study are included in the article/supplementary material, further inquiries can be directed to the corresponding author.

## Ethics Statement

The studies involving human participants were reviewed and approved by the Institutional Review Board, Alfaisal University, Riyadh, Saudi Arabia (Ref # IRB-20049). The patients/participants provided their written informed consent to participate in this study.

## Author Contributions

SAA and SAM: research conceptualization, manuscript writing, and manuscript editing. MA and LA: literature review. MI, JK, and AO: data collection and data checking and analysis. All authors have read and approved the manuscript.

## Funding

This work was supported by King Saud University, Riyadh, Saudi Arabia (RSP-2021/47).

## Conflict of Interest

The authors declare that the research was conducted in the absence of any commercial or financial relationships that could be construed as a potential conflict of interest.

## Publisher's Note

All claims expressed in this article are solely those of the authors and do not necessarily represent those of their affiliated organizations, or those of the publisher, the editors and the reviewers. Any product that may be evaluated in this article, or claim that may be made by its manufacturer, is not guaranteed or endorsed by the publisher.
